# Complication rates of percutaneous brachial artery puncture: effect of live ultrasound guidance

**DOI:** 10.1186/s42155-021-00262-2

**Published:** 2021-10-11

**Authors:** K. Appelt, M. Takes, C. J. Zech, KA Blackham, T. Schubert

**Affiliations:** 1 Radiology and Nuclear Medicine Clinic, University Hospital Basel, Basel, Switzerland; 2 Department of Neuroradiology, University Hospital Zurich, Zürich, Switzerland

**Keywords:** Brachial access, Ultrasound, Ultrasound-guidance, Endovascular, Intervention, Angiography

## Abstract

**Purpose:**

The current literature on the use of brachial artery access is controversial. Some studies found increased puncture site complications. Others found no higher complication rates than in patients with femoral or radial access. The purpose of this study was to determine the impact of ultrasound (US)-guidance on access site complications.

**Materials and methods:**

This is a single-center retrospective study of all consecutive patients with brachial arterial access for interventional procedures. Complications were classified into minor complications (conservative treatment only) and major complications (requiring surgical intervention). The brachial artery was cannulated in the antecubital fossa under US-guidance. After the intervention, manual compression or closure devices, both followed by a compression bandage for 3 h, either achieved hemostasis.

**Results:**

Seventy-five procedures in seventy-one patients were performed in the study period using brachial access. Access was successful in all cases (100%). Procedures in different vascular territories were performed: neurovascular (10/13.5%), upper extremity (32/43.2%), visceral (20/27.0%), and lower extremity (12/16.3%). Sheath size ranged from 3.2F to 8F (mean: 5F). Closure devices were used in 17 cases (22.7%). In total, six complications were observed (8.0%), four minor complications (5.3%, mostly puncture site hematomas), and two major complications, that needed surgical treatment (2.7%). No brachial artery thrombosis or upper extremity ischemia occurred.

**Conclusion:**

Exclusive use of US-guidance resulted in a low risk of brachial artery access site complications in our study compared to the literature. US-guidance has been proven to reduce the risk of access site complications in several studies in femoral access. In addition, brachial artery access yields a high technical success rate and requires no additional injection of spasmolytic medication. Sheath size was the single significant predictor for complications.

## Introduction

Since the advent of endovascular procedures, transfemoral access via the common femoral artery has been the preferred access site (Judkins 1967). However, since the first transradial access was described in 1989 by Campeau (Campeau 1989), there is a steady increasing approach of this technique, especially in cardiac- and recently also in neuro and body-interventions. In 2018 the American Heart Association (AHA) updated their recommendation to a “radial first” strategy due to level 1 evidence (Mason et al. 2018; Brueck et al. 2009) after randomized trials showed significantly reduced puncture site bleeding complications when using sheath sizes of up to 7 French (F), as well as reduced all-cause mortality (Valgimigli et al. 2015; Jolly et al. 2011). Additionally, ultrasound (US)-guidance was shown to improve the success rate of first-attempt arterial punctures while decreasing the time, as well as lowering the local complications such as hematomas from 3.4% to 1.4% (Shiloh et al. 2011; Seto et al. 2010a; 2010b).

However, transradial access can be limited not only by the vessel size prohibiting larger sheath placement than 7F but also the prolonged distance from the puncture site to the target area which may especially problematic when access to the abdominal or lower extremities is required (Chen and Peterson 2019). Moreover, radial artery spasm, radial or ulnar artery occlusion, as well radial artery tortuosity or anomalies can impede the transradial access (Mason et al. 2018; Seto et al. 2015; Pancholy et al. 2016) The major complication rate of radial arterial access is described as low as 0.5%, whereas the crossover rate is 4.9% (Burzotta et al. 2012).

The current literature on the use of a brachial access is controversial (Benit et al. 1997; Alvarez-Tostado et al. 2009). Whereas some studies found increased puncture site complications from 7 to 11% (Watkinson and Hartnell 1991; Grollman and Marcus 1988) up to 36% (Benit et al. 1997; Alvarez-Tostado et al. 2009), mainly consisting of bleeding complications and pseudoaneurysms (Watkinson and Hartnell 1991; Grollman and Marcus 1988; Heenan et al. 1996; Armstrong et al. 2003; Stavroulakis et al. 2016), other studies found no higher complication rates than in patients with femoral of transradial access (Grollman and Marcus 1988; Heenan et al. 1996).

Therefore, the aim of this study was to determine the impact of US-guidance on the rate of access site complications in a consecutive cohort of patients with brachial access in our institute. We hypothesized that the rate of complications will be lower than in comparable studies without US-guidance and that brachial access can be used for a wide variety of endovascular interventions successfully and safely.

## Methods

### Data collection

This is a single-center retrospective review. From January 2009 until January 2021, all patients who underwent an angiogram via brachial arterial access were reviewed. The local ethics committee of the University of Basel approved the study. All study protocols and procedures were conducted in accordance with the Declaration of Helsinki.

Data were extracted from the radiological information system (RIS) and the medical charts of the patients and included patient demographics, interventional body area, interventional technique, sheath and catheter size, peri- and post-interventional complications, as well as major adverse events, mainly death within 10 days.

Complications were further classified into minor complications (conservative treatment only) and major complications (requiring surgical intervention).

### Procedural details

The type of approach and puncture site was individually chosen by the interventional radiologist depending on the type of procedure.

The arm was extended on a specific arm board (STARSystem, Adept Medical) and the brachial artery was cannulated in the antecubital fossa with a micropuncture set (Radifocus® Introducer II Transradial Kit, Terumo) using a 22G Needle and a 0.18 in. wire after local anesthetic infiltration under live ultrasound-guidance. Spasmolytic agents were not applied, as mostly been using in radial access. If necessary, a larger sheath was subsequently inserted in Seldinger technique.

After completion of intervention, hemostasis was either achieved by manual compression or closure devices, followed by a compression bandage for 3 h. The decision was made by the interventionalist, based on various factors like puncture site, vessel size, sheath size and experience. Postprocedural evaluation of the puncture site and the peripheral perfusion was performed by default 1, 2, 3, and 6 h after finishing the procedure.

### Follow up

The follow up of the patients was performed by the attending disciplines. To assess the patient history, all the clinical data from the time of the intervention until 1 year after were investigated.

### Statistical analysis

For the statistical analysis, major and minor complications were grouped. Continuous variables are presented as means ± standard deviation, while categorical data are given as the counts (percentages). For the response variable complication (y/n), a general linear model was fit with anticoagulation (y/n), closure device (y/n) and sheath size (French, numeric) as predictors for which separate intercepts were fit. The level of significance was set at 0.05. The data were analyzed in r Project (“R Foundation”, Vienna, Austria).

## Results

### Data distribution

Seventy-five procedures via brachial artery approach in seventy-one patients were performed. The baseline demographics and patient characteristics can be seen in Table 1. One patient was excluded from the study because of death in the peri-operative period of his underlying disease, due to missing clinical follow-up of access site complications. Fourteen procedures (18.7%) were emergencies. The interventional target area was diverse with nine different vascular territories (Table 2). In 58 of the interventions (77.3%), manual compression followed by a compression bandage was applied for hemostasis. In 17 interventions (22.7%), a closure device (*n* = 9 Angio-Seal® VIP, Terumo Corporation; *n* = 7 Mynx; CardinalHealth, *n* = 1 Starclose, Abbott) was utilized. Mean follow up was up to 3 months after the procedure.

**Table 1:** Baseline demographics and patientcharacteristics

Variables	Percentage or mean
Age, y	66.9 (range 30 to 93)
Female	38 (51.4%)
Sheath size	5F (range 3F to 8F)
Major Complications	2.7%
Minor Complications	5.4%

**Table 2:** Treated vascular territories

Arterial region treated	Distribution
Upper Extremity retrograde	25
Visceral	15
Head and Neck	10
Upper Extremity antegrade	7
Pelvis	6
Lower extremity	6
Kidney	4
Bronchial arteries	1
Aorta	1

### Complications

In total, six complications were observed, four minor (5.3%) and two major (2.7%) complications (Tables 3 and 4).

**Table 3:** In total six complications occurred in 74 procedures

Complication	Occurrences	Treatment
Hematoma	3	Conservative
Pseudoaneurysm	2	One surgically, one conservative
Access site infection	1	Surgically
Bleeding	0	
Arterial thrombosis	0	
Nerve injury	0	
Unable to reach lesion	0

**Table 4:** The six complication in more details

	Complication	Procedure	Sheath size	Hemostasis	Consequence
**Major**	abscess	neurovascular	6F	Mynx	Surgical treatment
**Major**	pseudoaneurysma	visceral	6F	manual	Surgical treatment
**Minor**	pseudoaneurysma	lower extremity	6F	manual	conservative (ultrasound compression)
**Minor**	hematoma	upper extremity	6F	manual	conservative
**Minor**	hematoma	visceral	4F	manual	conservative
**Minor**	hematoma	lower extremity	6F	manual	conservative

The four minor complications included three hematomas at the puncture site, which were treated conservative without blood transfusion, and one pseudoaneurysm, which could be successfully treated with ultrasound-guided compression.

The first major complication was a surgically treated pseudoaneurysm. In this case, a 6F sheath was used and manual compression for bleeding control at the access site. The second major complication consisted of an abscess at the puncture site that required surgical drainage. In this case, a 6F sheath was used, and a 6F Mynx closer device.

### Statistical analysis

The estimate, standard error, z-value and *p*-value are summarized in Table 5. According to the general linear model, none of the predictors reached statistical significance. However, sheath size showed a *p*-value of 0.084 (two-tailed hypothesis). For a one-tailed hypothesis, the *p*-value for the predictor sheath size was 0.04. Mean sheath size in all complications was 5.66F (range: 4F - 6F), mean sheath size for the remaining cohort was 4.86F (range 2.7F - 8F).

**Table 5:** General linear model with anticoagulation (y/n), closure device (y/n) and sheath size (French, numeric) as predictors for which separate intercepts

Coefficients	Estimate	Std. Error	z value	*p*-value
Intercept	7.36	3.32	2.22	0.026 *
Anticoagulation (y/n)	0.28	0.96	0.3	0.76
Closure device	1.46	1.24	1.17	0.24
Sheath size	−0.86	0.5	−1.73	0.084

## Discussion

**Fig. 1:**
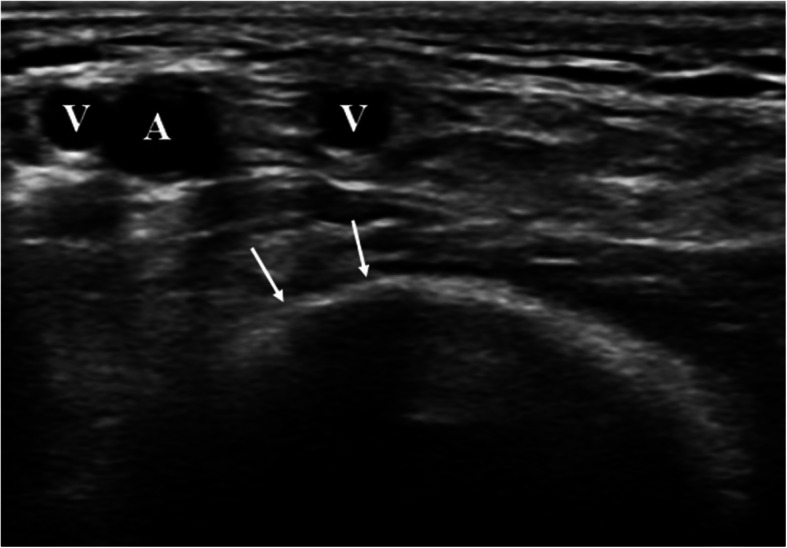
Transversal ultrasound picture of the A. brachialis (A) in the cubital fossa. The distal humerus (white arrows) forms an abutment, which helps for manual compressions. Two brachial veins (V) often accompany the distal A. brachialis

**Fig. 2:**
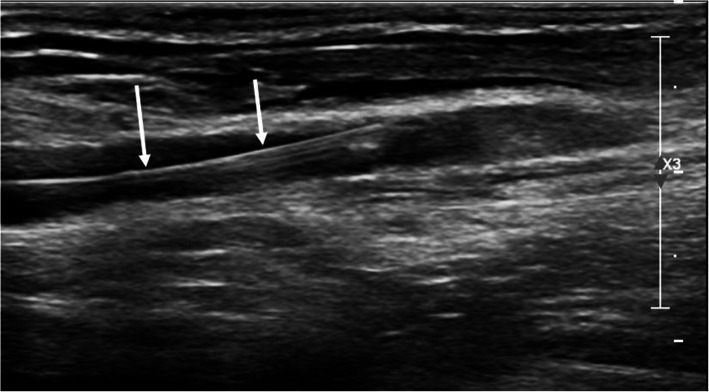
Longitudinal ultrasound picture of 19G puncture needle and guidewire (white arrows), which was inserted under live ultrasound guidance

In this retrospective study, we report our experience with ultrasound guided brachial artery access for endovascular procedures (Figs. 1 and 2). The major finding of the study is a low rate of access site complications using ultrasound guidance for brachial artery access. Especially, no case of upper extremity ischemia or brachial artery thrombosis occurred in our cohort. Larger sheath size was a significant predictor of complications. However, the application of a closure device did not result in a lower or higher complication rate.

**Fig. 3:**
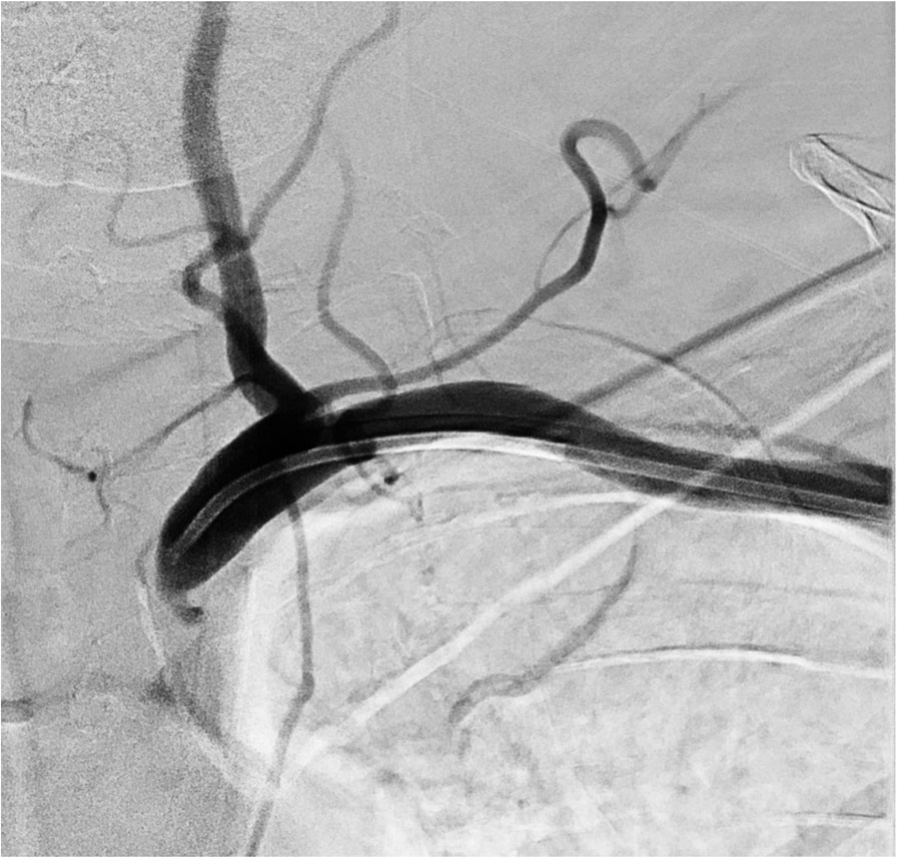
Digital subtraction angiogram (DSA) of a high-grade subclavian stenosis, treated with retrograde brachial access

Our results indicate that the consistent use of ultrasound guide for brachial artery access results in a low number of access site complications (Fig. 3). Especially major complications that require surgical intervention appeared at a low rate of 2.7%. Moreover, brachial artery thrombosis resulting in upper extremity ischemia did not occur in our series. We hypothesize that with US-guidance, the brachial artery is punctured at the optimal location in proximity to the bony landmark of the medial humeral condyle away from arterial bifurcations. This leads to an optimal compressibility against the medial humeral condyle after removal of the sheath.

It has been reported that brachial access is associated with a higher degree of access-related morbidity compared to femoral and radial access (Watkinson and Hartnell 1991; Stavroulakis et al. 2016; Madden et al. 2019; Franz et al. 2017). Overall complication rates as high as 36% have been described for brachial arterial access with major complications (hematoma, thrombosis, pseudoaneurysm, arteriovenous fistula, permanent neurologic deficit, and dissection) as high as 7% to 11% (Alvarez-Tostado et al. 2009; Armstrong et al. 2003; Franz et al. 2017).

However, recent studies have shown that BA access can be a safe and effective alternative to femoral access, with complication rates of between 1.3% and 3.4% reported (Franz et al. 2017).

One of these studies showed, that access-related complications increase with sheath size (Kret et al. 2016). This finding could be reproduced in our series.

The major complication rate in our patients was 2.7% (2 patients out of 74 procedures). No permanent deficit resulted from both complications which could be surgically resolved (one pseudoaneurysm, one access site abscess).

The effect of live ultrasound guidance has not been assessed well for brachial artery access, however, several studies demonstrated a significant lower access site complication rate at the femoral artery when using live ultrasound guidance (Shiloh et al. 2011; Seto et al. 2010a; Seto et al. 2015; Inagaki et al. 2018). The data review of our last 12 years of ultrasound-guided brachial access demonstrated a 100% access success rate, similar to the literature (Gan et al. 2010).

For access site closing, manual compression was used in 76% of cases. In the remaining 24%, vascular closing devices were utilized (Angio-Seal® VIP, Terumo Corporation, Tokyo / Mynx, and Starclose, Abbott). Currently, no vascular closing devices are intended for use in the brachial artery. However, some studies which reviewed off-label use in the brachial artery indicate these are likely safe (Lupattelli et al. 2008; Mirza et al. 2014). That coincides with our experience. No case of closing device failure or complications related to the closing device occurred in our series.

Arterial access at the upper extremity allows earlier mobilization compared with femoral access improving patient comfort (Armstrong et al. 2003; Franz et al. 2017). The advantages of brachial artery access compared to radial access are the bigger dimension of the puncture site vessel, as well as that standard catheter material can be used, in contrast to radial access, where shaft length of more than 100 cm is needed) and material for treatment of e.g. femoral lesions might be problematic even with 150 cm devices (Franz et al. 2017). Spasmolytics are generally not necessary for brachial access. Hence, in emergency situations, brachial artery access is swiftly achieved.

### Limitations

This review has some limitations. This a retrospective, nonrandomized study. The access site choice was by interventionalist preference.

Additionally, our study was limited by its relatively small sample size. We believe that additional studies with larger sample sizes are necessary, to confirm our low complication rates.

Our overall complication rate of 5.4%, with 2.7% requiring surgical intervention, is certainly comparable to standard femoral access, which is reported to be between 1.3% and 3.4% (Derubertis et al. 2007; Black et al. 2005; Archbold et al. n.d.; Piper et al. 2003). However, further studies of brachial artery access should be compared with a matched control group of femoral access.

## Conclusions

Our 12-year review of brachial access under live ultrasound guidance demonstrated that brachial access is a safe and reliable alternative to radial and femoral artery access. It offers a wide variety of endovascular interventions in every major peripheral arterial region. Live ultrasound guidance facilitated successful arterial access and reduced clinical complications. Future prospective and randomized studies could be completed to confirm its low complication rate, to be able to benefit from primary brachial arterial access.
